# Uterine diseases in cattle after parturition

**DOI:** 10.1016/j.tvjl.2007.12.031

**Published:** 2008-04

**Authors:** I. Martin Sheldon, Erin J. Williams, Aleisha N.A. Miller, Deborah M. Nash, Shan Herath

**Affiliations:** Department of Veterinary Clinical Sciences, Royal Veterinary College, Royal College Street, London NW1 0TU, UK

**Keywords:** Bovine, Uterus, Ovary, Disease, Immunity

## Abstract

Bacterial contamination of the uterine lumen is common in cattle after parturition, often leading to infection and uterine disease. Clinical disease can be diagnosed and scored by examination of the vaginal mucus, which reflects the presence of pathogenic bacteria such as *Escherichia coli* and *Arcanobacterium pyogenes*. Viruses may also cause uterine disease and bovine herpesvirus 4 (BoHV-4) is tropic for endometrial cells, causing a rapid cytopathic effect. The elimination of pathogens by the innate immune system is dependent on pattern recognition receptors binding pathogen-associated molecules. Uterine epithelial and stromal cells express receptors such as Toll-like Receptor 4 that binds *E. coli* lipopolysaccharide. The infertility associated with uterine disease is caused by damage to the endometrium and disruption of ovarian cyclic activity. Bacteria modulate endometrial prostaglandin secretion, and perturb ovarian follicle growth and function. Understanding the molecular basis of uterine disease will lead to novel approaches to treating infertility.

## Introduction

**Fig. 1:**
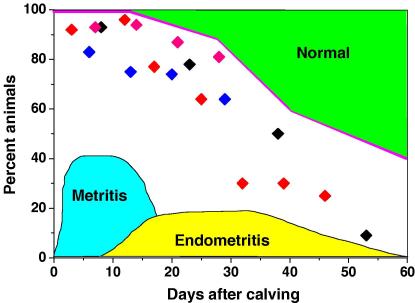
Almost all cows have bacteria within the cavity of the uterus during the first 2 weeks of calving and uterine disease is very common. Each marker () indicates the percent of animals with bacteria isolated from the uterine cavity; data are from four different studies, with animals sampled at various times between calving and 60 days after parturition (Elliot et al., 1968; Griffin et al., 1974; Sheldon et al., 2002b; Williams et al., 2005). The shaded areas represent the proportion of animals with metritis () within 2 weeks of calving and endometritis () 3–5 weeks after calving. The solid line () indicates the percent of animal with histological evidence of inflammation of the endometrium (Gilbert et al., 2005).

Parturition is a period of high risk for mother and offspring in all species, and cattle are no exception. As well as the risks of physical damage during the birth process or failure to release the placenta after parturition, there is often an upsurge of microbial infections in the cow. Some animals acquire infections of the uterus or mammary gland during late gestation, which may lead to premature parturition, or compromise fetal or calf health. However, the greatest impact on health and productivity is associated with microbial contamination of the uterine lumen after parturition. Amongst the mammals, *Bos taurus*, and particularly dairy cattle farmed in intensive systems, commonly acquire microbial contamination of the uterus. Indeed, 80–100% of animals have bacteria in their uterine lumen within the first 2 weeks after calving (Fig. 1). Although immune responses progressively eliminate the microbes, up to 40% of animals still have a bacterial infection 3 weeks after calving. Of course bacterial contamination does not always imply disease. The aim of the present review is to highlight the incidence, causes and consequences of uterine disease.

## Normal postpartum events

The events that must be completed after parturition before a cow is likely to conceive again are uterine involution, regeneration of the endometrium, elimination of bacterial contamination of the uterus and the return of ovarian cyclical activity. The initial stimulus for these changes to occur is the expulsion of the fetus along with the associated membranes and fluids at calving.

Uterine involution involves physical shrinkage, necrosis and sloughing of caruncles, and the regeneration of the endometrium. Following the loss of the allantochorion, there is necrosis of the uterine caruncles, which are usually sloughed by 12 days after parturition. Sloughing of the uterine caruncles contributes significantly to the rapid reduction in weight of the involuting postpartum uterus from 13 kg at parturition to about 1 kg 3 weeks later, because the caruncles account for over half of the weight of the uterus. The sloughed caruncles form the lochial discharge, along with the remains of fetal fluids and blood from the ruptured umbilicus. There is initially regeneration of the endometrium in the inter-caruncular areas and then by centripetal growth of the cells over the caruncle. Epithelial regeneration is complete by about 25 days after parturition, but the deeper layers of tissues are not fully restored until 6–8 weeks after calving.

The postpartum environment of the uterine lumen supports the growth of a variety of aerobic and anaerobic bacteria. Many of these bacteria are contaminants in the uterine lumen and are removed by a range of uterine defence mechanisms. However, uterine disease is commonly associated with *Escherichia coli, Arcanobacterium pyogenes, Fusobacterium necrophorum* and *Prevotella* species. Indeed, *A. pyogenes, F. necrophorum* and *Prevotella* species have been shown to act synergistically to enhance the likelihood of uterine disease, and increase the risk of clinical endometritis and its severity (Ruder et al., 1981; Olson et al., 1984). Numerically the most prevalent pathogens are *E. coli* (37% of pathogenic bacteria isolated) and *A. pyogenes* (49%) (Williams et al., 2005). Furthermore, the *E. coli* infections appear to precede and pave the way for the *A. pyogenes* infection (Williams et al., 2007).

After parturition, steroid hormone concentrations decrease to basal values, and there is an increase in plasma follicle stimulating hormone (FSH) concentration within days of calving that stimulates the emergence of the first postpartum follicular wave. Subsequently, the first dominant follicle is selected around 10–12 days after calving (Savio et al., 1990; Beam and Butler, 1997). These events occur in all postpartum cows irrespective of periparturient disease, environment or dietary deficiencies. However, the first dominant follicle has three possible fates: ovulation and formation of the first postpartum corpus luteum (return of ovarian cyclical activity), atresia with the emergence of one or more follicular waves without ovulation (anoestrus), or formation of an ovarian follicular cyst (Beam and Butler, 1997). Early return of ovarian cyclical activity is generally accepted to be beneficial for subsequent fertility (Darwash et al., 1997). However, it is suggested that an early postpartum first ovulation in the presence of uterine infection can lead to pyometra with persistence of a corpus luteum in the presence of pus within the uterine lumen (Olson et al., 1984).

## Incidence of uterine disease

The placenta is normally expelled within 6 h of expulsion of the calf but if still present by 24 h, it is defined as a retained placenta. The incidence of retained placenta is between 2% and 5% of animals in a herd, but can be increased in cows with twins, after dystocia and where infectious agents are endemic. The expression of clinical uterine infection depends on the balance between factors such as the animal, immunity, the number and pathogenicity of the microbes, and the uterine environment. Typically, 25–40% of animals have clinical metritis in the first 2 weeks after calving, and disease persists in up to 20% of animals as clinical endometritis (Fig. 1).

Although the clinical signs of uterine disease such as purulent material discharging from the uterus into the vagina are readily detected, the role of subclinical uterine disease is less well characterised but is an emerging issue. Up to 50% of cows 40–60 days after calving had neutrophils in the uterine lumen or endometrium, concomitant with inflammation of the tissues, and subclinical endometritis reduces conception rates (Kasimanickam et al., 2004; Gilbert et al., 2005). Chronic endometrial scarring, obstruction of the uterine fallopian tubes and adhesions between the ovary and the bursa are other consequences of uterine bacterial infection. However, these are less of a problem in cattle than other mammals including humans, with the incidence of ovaro-bursal adhesions affecting about 2% of cows.

## Definitions of uterine diseases

The definitions of uterine diseases encountered in cattle have been reviewed and articulated in Sheldon et al. (2006). Although it is not possible to categorise every animal with disease, the vast majority can be identified using these definitions. The definitions are repeated here for consistency but the original article should be consulted for the detail behind the definitions (Sheldon et al., 2006). *Puerperal metritis* is defined as an animal with an abnormally enlarged uterus and a fetid watery red-brown uterine discharge, associated with signs of systemic illness (decreased milk yield, dullness or other signs of toxaemia) and fever >39.5 °C, within 21 days after parturition. Animals that are not systemically ill, but have an abnormally enlarged uterus and a purulent uterine discharge detectable in the vagina, within 21 days after calving, may be classified as having *clinical metritis. Clinical endometritis* is characterised by the presence of purulent (>50% pus) uterine discharge detectable in the vagina 21 days or more after parturition, or mucopurulent (approximately 50% pus, 50% mucus) discharge detectable in the vagina after 26 days. In the absence of clinical endometritis, a cow with *subclinical endometritis* is defined by >18% neutrophils in uterine cytology samples collected 21–33 days after calving, or >10% neutrophils at 34–47 days. *Pyometra* is defined as the accumulation of purulent material within the uterine lumen in the presence of a persistent corpus luteum and a closed cervix.

In particular it is important to differentiate animals with metritis from those with endometritis. Metritis is infection of the cavity, lining and deeper layers of the uterus. On the other hand, endometritis is a localised infection of the lining of the uterus, which is inflamed with white pus mixed with mucus discharging from the uterus into the vagina. The deeper layers of the uterus are not affected by endometritis, so the uterus is not much bigger than that of a normal animal. Clearly, metritis is a much more severe disease than endometritis, requiring a different therapeutic approach. Firstly, it is much more urgent to identify cows with metritis promptly and, secondly, these animals need systemic treatments to counter the uterine infection and alleviate the generalized ill-health.

The use of the term pyometra should also be differentiated from clinical endometritis. Pyometra implies accumulation of pus within the uterine lumen associated with a closed cervix and a corpus luteum. There is often a corpus luteum present in animals with endometritis but the cervix is patent, often with pus discharging from the uterus into the vagina. In our experience, clinical endometritis is common whilst pyometra is relatively rare, comprising <5% of clinical cases of uterine disease. Fortunately treatment with prostaglandin (PG) F_2α_ is equally effective in both cases.

## Cause of uterine disease

Normal expulsion of the placenta involves three components that act in concert: (1) placental maturation associated with the endocrine changes in late pregnancy and around parturition (2) exsanguination of the fetal side of the placenta allowing shrinkage and collapse of the villi with separation from the crypts, and (3) uterine contractions with distortion of the placentomes. Why the placenta is retained for more than 24 h in some animals remains unclear and indeed there are likely to be several causes. As well as the more obvious causes of retained placenta such as disruption of normal parturition, including abortion, dystocia, twins and effects of diet, there may be genetic or immunological components to the problem associated with expression of major histocompatibility complex (MHC) molecules (Joosten et al., 1991).

The risk factors for uterine infection include retention of the placenta, the calving environment, twins, dystocia, and diet. Retained placenta is a particularly important predisposing factor for uterine infection (Odds ratio = 31–33, *P* < 0.001; T. Potter, J. Alcock, unpublished data). Furthermore, retained placenta is associated with a substantial reduction in milk yield that persists even after resolution of the problem and in one study affected animals (*n* = 13) produced 355 L less milk than normal cows (*n* = 77) during the first 60 days of lactation (Sheldon et al., 2004). The spontaneous rate for retention of the placenta is about 2–5%, but in about a quarter of the herds the retention rate is higher. Perhaps surprisingly, the microbial contamination of the calving environment is less well established as a predisposing factor for infection of the uterine lumen. However, it is important to note that there is bacterial contamination, clearance and re-contamination of the uterine lumen during the first few weeks after calving, not just infection around the time of parturition.

Unfortunately, little progress has been made toward the control or prevention of retained placenta or uterine disease. On the other hand, treatment protocols for these conditions are well established and reasonably effective; although, even after the resolution of the clinical signs there is still sub-fertility. Future progress is likely to depend on understanding the balance between infection and immunity in the female genital tract.

The uterine immune response to microbes leads to an influx of neutrophils from the peripheral circulation into the endometrium and uterine lumen (Zerbe et al., 2000, 2003; Dhaliwal et al., 2001). There are many natural antimicrobial peptides expressed in the genital tract, as well as acute phase proteins that also contribute to the defence of the uterus (Sheldon et al., 2001). Furthermore, the endometrial epithelial and stromal cells appear to have an immunological responsibility as they express pattern recognition receptors for the detection of microbes and produce a classical inflammatory response to bacteria (Herath et al., 2006b). However, bacteria or their products also modulate the normal endocrine function of these uterine cells (see below), which likely impacts not only the ability of the uterus to support an embryo but also affects ovarian function. Indeed, uterine disease is associated with extended luteal phases and failure to ovulate.

Conversely, the endocrine environment is likely to modulate uterine immunity. It has been known for more than 50 years that the risk of uterine infection is greater during the luteal phase of the oestrous cycle (Rowson et al., 1953a,b) and induction of luteolysis and oestrus is one of the most effective treatments for uterine infection (Sheldon and Noakes, 1998; Heuwieser et al., 2000; LeBlanc et al., 2002b). The mechanisms underlying this clinical dogma remain elusive but, at least in part, the effects on immunity may be associated with regulation of neutrophil function in the postpartum cow. In addition, dietary factors such as anti-oxidants and energy balance are likely to be important for neutrophil function and the immune response. On the other hand, ovarian steroids also modulate the response of the uterine cells to microbes (Herath et al., 2006b).

The role of viruses in uterine disease is relatively unexplored, although bovine herpesvirus 4 (BoHV-4) has been isolated from several outbreaks of metritis. In vitro*,* BoHV-4 is tropic for endometrial cells and efficiently infects epithelial and particularly stromal cells, causing a strong cytopathic effect (Donofrio et al., 2007). After viral entry into endometrial cells there was enhanced transactivation of the BoHV-4 immediate early gene promoter, which drives viral replication. Bovine macrophages are persistently infected with BoHV-4 and co-culture with endometrial stromal cells reactivated BoHV-4 replication in the macrophages, suggesting a symbiotic relationship between the stromal cells and the virus (Donofrio et al., 2007). This might be associated with stromal cell secretion of PGE_2_, which is known to reactivate BoHV-4 replication in persistently infected macrophages (Donofrio et al., 2005).

## Diagnosis of uterine disease

The examination of the contents of the vagina for the presence of pus is the most useful procedure for diagnosis of uterine infection (Bretzlaff, 1987; LeBlanc et al., 2002a; Williams et al., 2007). The usual method for assessing the contents of the vagina is to perform a manual examination. This technique is cheap and rapid, whilst providing additional sensory information such as the detection of vaginal lacerations. One procedure is to clean the vulva using a dry paper towel and insert a clean, lubricated gloved hand through the vulva into the vagina and withdraw the mucus contents of the vagina for examination. Manual vaginal examination does not cause uterine bacterial contamination, provoke an acute phase protein response, or affect uterine horn diameter (Sheldon et al., 2002a). Other options are to use vaginoscopy, using autoclavable plastic or disposable foil-lined cardboard vaginoscopes, to visualise the mucus flowing out of the cervix, or to use an instrument to withdraw the vaginal contents.

The character and odour of the vaginal mucus can be scored to produce a clinical endometritis score. A mucus character score is assigned between 0, clear translucent mucus; 1, clear mucus containing flecks of white pus; 2, exudate containing ⩽50% white or cream pus; 3, exudate containing >50% white, cream or bloody pus (www.rvc.ac.uk/endoscore/) (Sheldon et al., 2006).

The vaginal mucus odour is scored 0 for no odour and 3 if a fetid odour is present. The character and odour scores are summed to give an endometritis clinical score ranging from 0 to 6. Although few animals with a mucus character score of <3 also have a fetid odour, weighting the fetid odour score as 3 avoids the potential confusion that might occur if the score was 1. The clinical endometritis score reflects the presence and semi-quantitative load of recognised uterine pathogens, but not other bacteria in the uterine lumen (Williams et al., 2005). For example, using a semi-quantitative method the median bacterial growth density of pathogenic bacteria in the uterine lumen was higher for animals with purulent (4 vs. 1, *P* < 0.05) or fetid (4 vs. 1, *P* < 0.05) material in the vagina than normal animals. In addition, endometritis clinical score is prognostic for the likely success of treatment (Sheldon and Noakes, 1998). The success rate for cure of endometritis over a 2 week period, as determined by achieving a final vaginal mucus score of 0, was 44% if the vaginal mucus was purulent with a fetid odour at the start of treatment, but 78% if there were only flecks of pus in the mucus.

## Consequences of uterine disease

Clinical and subclinical uterine diseases are associated with sub-fertility and infertility. At the herd level this is characterised by longer intervals from calving to first insemination or conception for affected animals, and more cows culled for failure to conceive in a timely manner (Kossaibati and Esslemont, 1997; Esslemont and Kossaibati, 2002). These effects on fertility and the costs of treatment mean that uterine disease is one of the most expensive conditions challenging the dairy industry. Furthermore, the high incidence of uterine disease in cattle compared with other domestic species suggests that there may be critical flaws in dairy cow husbandry or a fundamental problem with some breeds of cow. Thus, considerable effort needs to be made to understand the risk factors for uterine disease and the biological mechanisms underlying how the uterus is able to detect infection, respond to the microbes and how infection modulates normal uterine function.

In a typical study the first service conception rate was lower for cows with endometritis (29.8% vs. 37.9%), the median calving to conception interval was longer (151 vs. 119 days) and there were more animals culled for failure to conceive (6.7% vs. 3.8%), than unaffected animals (LeBlanc et al., 2002a). Similarly, cows with a purulent cervical discharge have lower submission rates, lower pregnancy rates and more culls for failure to conceive (McDougall, 2001). The financial losses associated with uterine infection are dependent on the cost of treatment, reduced milk yield, and infertility. In the UK, the direct costs of treatment and reduced milk yield of a cow with uterine disease are about €91^1^ and uterine disease results in an average lactation loss of about 300 L (Esslemont and Kossaibati, 2002). The indirect costs of a longer calving interval, increased culling rate, extra inseminations and lower oestrus expression are €101 per cow, giving a total cost of €192 per animal. The direct costs of uterine disease alone were estimated to be €1059 per 100 cows each year using information from 21 dairy herds in the UK where data were recorded intensively between 1989 and 1999. There were 19.5 × 10^6^ dairy cows in the EU15, and projecting the direct costs of uterine disease on that basis would represent a cost of more than €206 million. Of course, the indirect costs of disease would more than double this amount.

## Mechanisms underlying uterine disease and infertility

Uterine disease such as retained placenta and uterine infection are key risk factors for the occurrence of abnormal progesterone profiles indicating delayed ovulation, cystic ovarian disease or long luteal phases (Opsomer et al., 2000; Royal et al., 2000). In our experience, cows with uterine disease were less likely to ovulate the first postpartum dominant follicle (8% vs. 40%, *P* < 0.05; E. Scarr, unpublished data) and more likely to have abnormal progesterone profiles (58% vs. 39%, *P* < 0.05; J. Sykes unpublished data) than normal animals. These effects of uterine disease on ovarian function are likely mediated at multiple levels: ovary, hypothalamus and pituitary. Similarly, the low conception rates in cattle with subclinical endometritis or after resolution of other uterine disease are probably the consequence of disruption of endocrine pathways and physiology as well as associated with uterine inflammation.

### Uterine function

It is assumed that a healthy endometrium is necessary for the nutrition of the blastocyst and embryo, and the successful establishment of pregnancy. Certainly infection with pathogenic bacteria appears to preclude conception. Furthermore, there is embryo mortality if uterine infection occurs with these bacteria after conception (Semambo et al., 1991). Viruses such as bovine virus diarrhoea virus have a similar effect (McGowan et al., 1993).

As the uterus is usually sterile, the presence of microbes or pathogen-associated molecules appears to provoke a substantial immune response. The uterine immune response is generated by immune cells within the endometrium and by the endometrial stromal and epithelial cells. Indeed, it is the epithelial cells that are the first line of defence against microbes in the uterine lumen.

Innate immunity in the genital tract is highly dependent on the expression of pattern recognition receptors (PRRs) to detect pathogen-associated molecular patterns (PAMPs). These PRRs, such as the family of Toll-like Receptors (TLRs), are highly conserved across phyla and detect a range of PAMPs associated with fungi, viruses and bacteria (Beutler, 2004; Akira et al., 2006). Binding of PAMPs to PRRs activates signal transduction pathways for mitogen-activated protein kinase (MAPK) and the nuclear factor-kappa B (NFκB) transcription factors, leading to secretion of prostaglandins, cytokines and chemokines (Ghosh et al., 1998; Li and Verma, 2002; Akira and Takeda, 2004). Epithelial and stromal cells express toll-like receptor 4 (TLR4), the innate immune receptor for lipopolysaccharide (endotoxin, LPS), which is the key PAMP of the common uterine pathogen *E. coli* (Herath et al., 2006b). This concept of endometrial cell expression of PRRs is supported in other species by expression of other toll-like receptors for bacteria and viruses (Schaefer et al., 2004; Hirata et al., 2005; Soboll et al., 2006). However, bovine cell cultures have the advantage over other species because they are free of contamination with professional immune cells, as determined by the absence of CD45 expression (Herath et al., 2006b). The bovine TLR4 signalling pathways are also emerging (Connor et al., 2006). This makes the bovine system a useful model for exploring the mechanisms of uterine disease in other mammals as well as cattle (Herath et al., 2006a).

The effect of pathogen-associated molecules on uterine cells is not limited to inflammation, but also affects endocrine function. The principal hormones secreted by the endometrium are PGF_2α_ and PGE_2_, respectively, and the secretion of these hormones is modulated by *E. coli* or LPS (Herath et al., 2006b). The prediction from the in vitro work is that uterine disease would extend the luteal phase, which is what is observed clinically. Furthermore, exogenous PGF_2α_ is an effective treatment for uterine disease and eicosanoids may modulate uterine immunity directly (Lewis and Wulster-Radcliffe, 2006). In vitro, LPS stimulates progesterone secretion from mixed populations of luteal cells (including steroidogenic, endothelial and immune cell types) to a level similar to that seen with luteinising hormone (LH), but at higher concentrations LPS kills the cells (Grant et al., 2007).

### Ovarian function

A large healthy oestrogenic follicle at the time of ovulation is important for establishment of a successful pregnancy (Perry et al., 2005). However, as well as effects on luteal function, uterine infection also perturbs ovarian follicle growth and function (Sheldon et al., 2002b). Cows with uterine disease have smaller ovarian follicles and lower peripheral plasma oestradiol concentrations. Furthermore, this might be a localised effect of uterine infection on ovarian function because when uterine bacterial growth scores were high fewer first (1/20 vs. 15/50, *P* < 0.05) or second postpartum dominant follicles (1/11 vs. 13/32, *P* < 0.05) were selected in the ovary ipsilateral to the previously gravid uterine horn than the contralateral ovary (Sheldon et al., 2002b).

At the level of the hypothalamus and pituitary, the oestradiol-induced preovulatory LH surge is blunted when bacterial endotoxin is infused into the uterus or administered intravenously (Peter et al., 1989, 1990; Battaglia et al., 1997). Indeed, LPS or various intermediary cytokines such as interleukin (IL)-1 or tumour necrosis factor (TNF)-α block gonadotrophin releasing hormone (GnRH) secretion and the pituitary responsiveness to GnRH pulses (Rivest et al., 1993; Battaglia et al., 2000; Williams et al., 2001). However, uterine infection does not appear to affect peripheral plasma FSH concentration profiles, or ovarian follicle wave emergence.

## Conclusions

Retained placenta, uterine bacterial infection and uterine disease are common after parturition in cattle and cause considerable infertility. Uterine bacterial infection stimulates a robust immune response but also modulates normal reproductive physiology. Increasing knowledge about the interaction between the environment, uterine infection, immunity and reproduction should lead to improved control and treatment strategies for cattle infertility.

## Conflict of interest statement

Martin Sheldon has grant funding from veterinary pharmaceutical companies. None of the other authors (Erin J. Williams, Aleisha N.A. Miller, Deborah M. Nash, Shan Herath) has a financial or personal relationship with other people or organisations that could inappropriately influence or bias the paper entitled *Uterine diseases in cattle after parturition.*

## Footnotes

1 €1 = approx. £0.75, US$1.48, as at 24 February 2008.
